# Supplementary material for: Monoclonal Antibodies as SARS-CoV-2 Serology Standards: Experimental Validation and Broader Implications for Correlates of Protection
Source: Int J Mol Sci. 2023 Oct 28;24(21):15705. doi: 10.3390/ijms242115705 (PMC10650176; doi:10.3390/ijms242115705)
Supplement: Supplementary file 1 [file ijms-24-15705-s001.zip › ijms-2649228-supplementary.pdf]

Supplementary Materials

Detailed Study Design

A dry-ice package containing three sets of study materials allowing for three independent day measurements and one vial of the first WHO IS was shipped to each participant: Abbott Laboratories, FDA, Fredrick National Laboratory for Cancer Research (FNLCR), NIST, and Roche Diagnostics. Each set of study materials contained one vial for each of the three mAbs (30 µL aliquot, 2 mg/mL in PBS, and 0.05% NaN_3) and one vial of each de-identified, blinded convalescent plasma/serum sample (150 µL sample volume). It was recommended that each participant would reconstitute the vial of WHO IS with a 250 µL of sterile distilled water, split into three vials with an equal volume, and then place one vial with each set of the study materials.

The study protocol, provided to participants, specified the utilization of freshly thawed aliquots that must be used within the same day of thawing for each independent day measurement. For the 'Day 1' measurement, the participants were asked to collect results from the serial dilutions in two replicates for each sample type and determine an optimal dilution range with a minimum of five dilutions that covers at least one point beyond the endpoint dilution (baseline/background signal for the sample). Adjustment of sample dilutions could be made based on 'Day 1' results for the subsequent assay days (Day 2 and Day 3), if needed. Given the large number of samples, each 'Day' measurements on 62 serum/plasma samples, 3 mAb, and WHO IS could be split up and run over multiple consecutive or non-consecutive days. However, the use of in-house negative and positive assay controls was strongly encouraged to ensure the quality of assay readouts over multiple days.

A Microsoft Excel reporting spreadsheet was provided to harmonize the reporting of assay information and results. For each sample dilution, the participants were asked to record the raw assay readout (e.g., absorbance (OD) and mean fluorescence intensity (MFI)). The participants were encouraged to report the results in the unit of BAU/mL based on the calibration using WHO IS or ng/mL utilizing mAb with the known concentrations for the serological-binding assay and NT50 values for the neutralization assay, as per the participating laboratory's SOP. A cut-off value indicating seroreactivity for each assay and the status of whether each sample dilution tested is considered positive or negative (or indeterminate) were also included in the study's reporting spreadsheet.

Additional Details Related to the Normalization and Harmonization of the Serological-Binding Assay

On Units: Because normalization and harmonization are fundamental tasks in metrology, an important first step in our analysis was to establish conventions for representing antibody measurements. We define the dimensionless, scaled antibody concentration $\hat{y}_{s,n,r}$ of sample *s* relative to reference *r* using assay *n* as

$$\hat{y}_{s,n,r} = \frac{c_{s,n}}{c_{r,n}} \qquad (9)$$

where $c_{s,n}$ and $c_{r,n}$ are the absolute concentrations of bound antibodies associated with the sample and reference, respectively. Note that the reference is a generally a calibrator for the specific assay. Here and throughout the paper, the subscripts *s, n,* and *r* are reserved for a sample, assay, and reference, respectively.

As $\hat{y}_{s,n,r}$ is dimensionless, and convention dictates that we attach units to normalized concentrations, we manually assigned an arbitrary binding antibody unit (BAU) concentration *B*, e.g., associated with the WHO IS or another standard, and denoted the corresponding normalized antibody concentration by

$$\hat{c}_{s,n,r} = \hat{y}_{s,n,r}B \qquad (10)$$

Because binding antibody assays can be viewed as equilibrium chemical reactions associated with Equation (1), normalized concentrations are related to the associated Gibbs free energy $\Delta G_{s,n,r}$. This makes more appropriate to work with logarithms of $\hat{y}_{s,n,r}$, via

$$\gamma_{s,n,r} = \ln\left(\hat{c}_{s,n,r}/B\right) = \ln(\hat{y}_{s,n,r}) = -\Delta G_{s,n,r}. \qquad (11)$$

We refer the reader to [1] for more details, although we note that the equivalence of serology references discovered in this paper arises from a universal structure of $\Delta G_{s,n,r}$. Equation 4 also allows us to express the numerical value of the logarithm of $\hat{c}_{s,n,r}$ via

$$\ln\left(\hat{c}_{s,n,r}\right) = \gamma_{s,n,r} + \ln{(B)} \qquad (12)$$

provided that we use only the numerical part of $\hat{c}_{s,n,r}$ and *B* (and not their units) when evaluating the logarithms in Equation (5). (In a slight abuse of notation, we always interpret logarithms of quantities with dimensions as applying only to the numerical parts of the arguments. Note also that logarithms of *B* and $\hat{c}_{s,n,r}$ are themselves dimensionless.) Equations (9) and (11) remain the fundamental quantities whose estimation achieves normalization and harmonization, while Equations (10) and (12) are semantic choices associated with a different representation of normalized measurements. We henceforth refer to $\gamma_{s,n,r}$ and $\ln\left(\hat{c}_{s,n,r}\right)$ as the log-scaled concentrations and log-normalized concentrations, respectively, although the mathematical analysis uses primarily the former.

Finally, we note that harmonization can be performed using either the $\gamma_{s,n,r}$ or $\hat{c}_{s,n,r}$. For physical reasons, the former is a more natural quantity with which to work, and we defined the harmonized log concentration $\ln \chi_{s,r}$ in terms of $\gamma_{s,n,r}$. This induces the definition $\ln \chi_{s,r} = \ln(\tilde{c}_{s,r}/B)$ for the consensus value $\tilde{c}_{s,r}$, which has units of *B*.

Serological-Binding Result Summary: Raw data from each lab was provided as dilution–MFI or dilution–OD pairs across a range of dilutions. Each sample was measured at least once a day for three days, except in the case of one lab that only measured certain samples on two separate days. For each lab, if on a fixed day a sample–dilution pair was measured more than once, we averaged the corresponding measurements and used the resulting mean as the scalar measurement value associated with the sample–dilution–day complex. All normalization discussed in the next section was conducted using the reference measurements performed on the same day as the corresponding sample–dilution measurement. For each assay, we discarded fluorescence–dilution pairs for which the MFI falls outside of an assay-dependent saturation range as well as data from negative samples.

Normalization of the Serological-Binding Assays: For end-point titer analyses using only data in the linear range [2], the ratio of dilution-weighted MFI is equivalent to Equation 9, which is expressed as a ratio of concentrations. However, this approach does not necessarily take advantage of all available data and makes unnecessary assumptions about the instrumentation. This motivated us to develop a novel approach that generalizes the slope-correction method [3] and directly estimates the ratio of concentrations in Equation (9). As discussed in the next paragraphs, the ratio $\hat{y}_{s,n,r}$ can be estimated using our method, even though $c_{s,n}$ and $c_{r,n}$ are always unknown. The main idea behind our approach is to simply recognize that the measurement signal f (e.g., MFI) is an increasing function of the bound antibody number, and not the concentration, provided the reporter molecule (e.g., a fluorophore) does not exhibit self-quenching. This generally holds, irrespective of whether the dilution curve is linear or not. Moreover, the antibody number x associated with a sample is simply the concentration multiplied by the dilution factor d, viz., $x_{s,n} = c_{s,n}d$. Thus, we arrived at the conclusion that the function $f(x_{s,n}) = f(c_{s,n}d)$ is a rescaled version of $f(x_{r,n}) = f(c_{r,n}d)$, where the conversion factor

$$\alpha_{s,n,r}^{-1} = c_{s,n}/c_{r,n} = \hat{y}_{s,n,r} \quad (13)$$

accounts for horizontal dilation or contraction of $f(x_{s,n})$ relative to $f(x_{r,n})$. In other words, by dilating or contracting the dilution curve for a sample so that it collapses onto the reference, the factor associated with this transformation is the scaled antibody concentration. Note that estimating $\hat{y}_{s,n,r}$ in this way is tantamount to normalizing the concentration, since this quantity is equivalent to $\hat{c}_{s,n,r}$ via Equation (3). Moreover, the scaling factor $\alpha_{s,n,r}$ laterally translates the dilution curve (via a horizontal shift instead of contraction or dilation) when considering fluorescence as a function of $\gamma_{s,n,r} = \ln(\hat{y}_{s,n,r})$. We refer the reader to [1] for more details.

In practice, estimating the $\alpha_{s,n,r}$ in this way is complicated by the fact that typical dilution series are only measured at a few dilutions. We therefore developed a constrained optimization approach that simultaneously estimates all $\alpha_{s,n,r}$ along with a "master curve" that is a dense representation of the dilution curve for the reference material [1]. Importantly, this analysis can work with fluorescence signals that do not display any linearity with concentration, but fully reduces to both the end-point titer and slope-correction methods when considering only data in the linear part of a dilution curve. In our analysis, we use all available data provided it does not manifest self-quenching.

In this study, we independently estimated the $\gamma_{s,n,r}$ multiple times by applying our analysis to data collected over several days, the latter of which is described in the Data Summary Section. Denoting these normalized measurements $\gamma_{s,n,r,t}$ (where t indexes time or day), we took the arithmetic mean over t as our final or average log-scaled antibody concentration $\bar{\gamma}_{s,n,r}$ for use in all later calculations. The arithmetic mean of log concentrations is equivalent to a geometric mean of the normalized concentrations. The justification for this choice is based on the physics of Gibbs free energies [1].

Harmonization of the Serological-Binding Assays: Through thermodynamic justifications [1], harmonization proceeds by recognizing that the averaged log antibody concentration $\gamma_{s,n,r}$ associated with sample s, assay n, and reference r can be expressed in the form

$$\bar{\gamma}_{s,n,r} = \ln(c_{s,r}) + \Delta g_{r,n} - \Delta g_{s,n} + \delta_{s,n} \qquad (14)$$

where $c_{s,r}$ is the dimensionless consensus antibody concentration associated with the sample and a reference, and $\Delta g_{r,n}$ and $\Delta g_{s,n}$ are free energies associated with the reference–assay pair and sample–assay pair, respectively. The term $\delta_{s,n}$ are "within-lab" uncertainties estimated from repeated measurements; see the main text for additional discussion. Equation 14 induces the following definition of a harmonized measurement $H_{s,r}$, with a corresponding consensus value $\chi_{s,r}$:

$$\ln(H_{s,r}) = \bar{\gamma}_{s,n,r} - \Delta g_{r,n} = \ln(c_{s,r}) - \Delta g_{s,n} + \delta_{s,n} \, . \quad (15)$$

When equipping the consensus value with units, we refer to it by the symbol $\tilde{c}_{s,r}$, which is the normalized analog of the scaled $\chi_{s,r}$, that is, $\tilde{c}_{s,r} = \chi_{s,r} B$ for B units. Importantly, the $\Delta g_{r,n}$ is an assay-dependent bias, whereas we treated $\Delta g_{s,n}$ as random or residual bias, since the specific sample–assay interactions are unknown a priori. Our companion paper derives the thermodynamic relationships proving that the effect $\Delta g_{r,n}$ of the reference material on the consensus value is sample-independent and ultimately does not affect estimation of $\chi_{s,r}$ and $\tilde{c}_{s,r}$ when our analysis method is used.

To determine the consensus values for all samples, we posited that the $\Delta g_{s,n}$ are sample-dependent realizations of a normal random variable with an unknown, assay-dependent variance ς_n^2. Given the

average values $\bar{\gamma}_{s,n,r}$ and corresponding standard deviation $\sigma_{s,n,r}$ (associated with $\delta_{s,n}$), we used a regularized maximum likelihood (MLE) analysis to determine the unknown parameters in Equation 6, namely, the $\tilde{c}_{s,r}$, $\Delta g_{r,n}$, and variance ς_n^2 associated with $\Delta g_{s,n}$. These parameters were used to quantify, and ultimately remove, the variation in normalized measurements arising from the choice of reference. The harmonized measurements were then defined to be the bias-corrected difference $\bar{\gamma}_{s,n,r} - \Delta g_{r,n}$, which equals the consensus plus residual sample–assay-dependent uncertainty. We refer the reader to [1] for complete details, including a justification of the fact that $\delta_{s,n}$ is reference-independent.

Detailed Neutralization Assay Analysis

For surrogate neutralization assay, beads coated with RBD were incubated with dilutions of a serum sample containing nAb. Here, we assumed that the Hill equation does not work for this reaction as well as it worked for the serological-binding measurement. The binding of nAb to the beads with immobilized antigens can be described by the Hill equation, shown in Equation 7, with N_{nAb} replacing N_{Ab}, which models the antigen site occupancy at different dilutions of nAb. N_{max} and N_{min} are the maximum and minimum site occupancy on the microspheres, respectively, and N_{nAb} is the total number of antigen sites occupied by nAb at each dilution *dil*.

The fundamental variable is the site occupancy and, since there is no direct measurement of the number of nAb on the bead, we used the Hill equation to model this number. The total number of antigen sites was fixed, so that $N_{max} = N_{nAb} + N_{free}$, where N_{free} is the number of unoccupied sites on the bead. Next, the microspheres were incubated with biotinylated ACE2 proteins. The ACE2 coupled strongly to free RBD sites on the microspheres. Finally, the beads with the RBD–ACE2–biotin complex were incubated with PE–streptavidin, which couples strongly with biotin. The last two steps were conducted at saturated concentrations ACE-2–biotin and subsequently streptavidin–PE so that all the free RBD sites were coupled to ACE2–biotin–streptavidin–PE complex. We assumed that each free RBD site was coupled to one PE fluorescent protein, so that $N_{free} = N_{PE} = N_{max} - N_{nAb}$. Substituting Equation 7 for N_{nAb} reveals

$$N_{PE} = N_{max} - \left(N_{max} + \frac{N_{min} - N_{max}}{\left(1 + \left(\frac{NT_{50}}{dil}\right)^n\right)}\right) = \frac{N_{max} - N_{min}}{\left(1 + \left(\frac{NT_{50}}{dil}\right)^n\right)}$$

$$\frac{N_{PE}}{N_{max}} = \frac{1 - N_{min}/N_{max}}{\left(1 + \left(\frac{NT_{50}}{dil}\right)^n\right)}$$

The flow cytometer (FC) measures the fluorescence intensity of a single bead. Many beads are passed through the FC, and the analysis of the fluorescence pulses provides MFI and CV% for the bead

population at a specific dilution of the nAb sample used for binding the nAb on the bead. Assuming a linear cytometer response, the ratio of sites occupied by PE to the maximum number of such sites is equal to the ratio of PE fluorescence to the maximum PE fluorescence if all sites were occupied by PE. Therefore, performing some minor algebra leads to Equation 16.

$$\frac{N_{PE}}{N_{max}} = \frac{MFI_{PE}}{MFI_{PEmax}} = \frac{1-N_{min}/N_{max}}{(1+\left(\frac{NT_{50}}{dil}\right)^{n})} \quad (16)$$

Equation 16 expresses the fraction of sites unoccupied by nAb at each dilution. The result $1 - MFI_{PE}/MFI_{PEmax}$ shows the fraction occupied by nAb and thus not available to infect cells. In other words, it is the fractional reduction in available sites for infection. The final model equation for fitting reduction% data is shown below, in Equation 17.

$$\frac{MFI_{max}-MFI}{MFI_{max}} = reduction\% = 100(1 - \frac{1-N_{min}/N_{max}}{\left(1+\left(\frac{NT_{50}}{dil}\right)^{n}\right)}) \quad (17)$$

The ratio N_{min}/N_{max} is a parameter and 100 is the conversion from fraction to percentage. A similar argument justifiesd the analysis of measurements obtained with the pseudovirus and live virus. In both cases, it was assumed that the infection of cell is proportionate to the number of free sites on the virus (unbound by nAb).

Readout Characteristics of the Six Serological-Binding Assays: For each lab, data were reported in arbitrary MFI or OD units. The optimization algorithm used to normalize sample concentrations to each reference does not assume a linear range, but it does require that the fluorescence or absorbance be monotone increasing in concentration. We manually identified approximate bounds for each assay over which this condition is satisfied. Only data within the corresponding bounds for each assay were used in the normalization analysis.

Laboratory Number	Lower MFI/OD Bound	Upper MFI/OD Bound
**1**	30	90000
**2**	150	200000
**3**	0.095	0.25
**4**	0.095	0.25
**5**	1000	350000
**6**	0.3	3.1

Broader Implications for Serology: The conclusions in this manuscript point to several looming and related issues for the serology community. There does not yet appear to be a complete discussion, much less scientific consensus, on the purpose of harmonization and its connection to uncertainty quantification (UQ). In the broader metrology literature, both tasks are often performed to facilitate decision making and/or ensure an engineering system satisfies design requirements, for example, and this is the perspective we adopted. However, the relationship between binding and neutralization antibody titers may not be the best quantifying protection, or other more appropriate applications of serology may emerge, requiring different concepts of harmonization.

This points to the fact that we cannot determine a "best" standard without first establishing the context for and, thus, a definition of harmonization. In this work, that context is a diagnostic decision: does a binding level indicate a given neutralization level, and how certain are we in that conclusion? Through the induced concept of harmonization, this leads to the perspective that the technical performance of a standard is defined as the added uncertainty in consensus values associated with using a given reference. This is a quantitative metric that directly links the reference material back to its underlying context (decision making) and yields the result that all binding references are equivalent. But this perspective depends on how the standard is used, it stands to reason that one might find a unique "best" standard associated with different contexts.

These observations also point to the broader issue that technical performance and fitness-for-purpose may not be the same. The latter is clearly desirable, but to the best of our knowledge, it remains largely undefined in serology. In this paper, the equivalent performance of all references suggests that the fitness of a standard should consider not only technical performance, but also other factors, such as time-to-market and traceability. The task of identifying the considerations that inform fitness-for-purpose remains an open question beyond the scope of this manuscript.

References:

1. Patrone, P.N.; Wang, L.; Lin-Gibson, S.; Kearsley, A.J. Uncertainty Quantification of Antibody Measurements: Physical Principles and Implications for Standardization. **2023**, *in review*.
2. Frey, A.; Di Canzio, J.; Zurakowski, D. A statistically defined endpoint titer determination method for immunoassays. *J. Immunol. Methods* **1998**, *221*, 35–41.
3. Barrette, R.W.; Urbonas, J.; Silbart, L.K. Quantifying Specific Antibody Concentrations by Enzyme-Linked Immunosorbent Assay Using Slope Correction. *Clin. Vaccine Immunol.* **2006**, *13*, 802–805.
